# Atomically Dispersed Ruthenium Catalysts with Open Hollow Structure for Lithium–Oxygen Batteries

**DOI:** 10.1007/s40820-023-01240-0

**Published:** 2023-11-21

**Authors:** Xin Chen, Yu Zhang, Chang Chen, Huinan Li, Yuran Lin, Ke Yu, Caiyun Nan, Chen Chen

**Affiliations:** 1 Beijing Advanced Innovation Center for Materials Genome Engineering, Institute of Solid State Chemistry, University of Science and Technology Beijing, Beijing, 100083 People’s Republic of China; 2 Engineering Research Center of Advanced Rare Earth Materials, Department of Chemistry, Tsinghua University, Beijing, 100084 People’s Republic of China; 3 Beijing Key Laboratory of Energy Conversion and Storage Materials Institution, College of Chemistry, Beijing Normal University, Beijing, 100875 People’s Republic of China

**Keywords:** Atomically dispersed, Open hollow structure, Discharge product, Lithium, Oxygen battery

## Abstract

Lithium–oxygen battery with ultra-high theoretical energy density is considered a highly competitive next-generation energy storage device, but its practical application is severely hindered by issues such as difficult decomposition of discharge products at present. Here, we have developed N-doped carbon anchored atomically dispersed Ru sites cathode catalyst with open hollow structure (h-RuNC) for Lithium–oxygen battery. On one hand, the abundance of atomically dispersed Ru sites can effectively catalyze the formation and decomposition of discharge products, thereby greatly enhancing the redox kinetics. On the other hand, the open hollow structure not only enhances the mass activity of atomically dispersed Ru sites but also improves the diffusion efficiency of catalytic molecules. Therefore, the excellent activity from atomically dispersed Ru sites and the enhanced diffusion from open hollow structure respectively improve the redox kinetics and cycling stability, ultimately achieving a high-performance lithium–oxygen battery.

**Supplementary Information:**

The online version contains supplementary material available at 10.1007/s40820-023-01240-0.

## Highlights

- The open hollow structure enhances the accessibility of atomically dispersed Ru sites and improves the diffusion efficiency of catalytic molecules
- The abundant atomically dispersed Ru sites on the surface of catalyst provide a large number of oxygen adsorption and Li_2_O_2_ nucleation sites
- The lithium–oxygen battery assembled with h-RuNC could catalyze the production of Li_2_O_2_ with better dispersion and improve the cycling stability.

**Supplementary Information:**

The online version contains supplementary material available at 10.1007/s40820-023-01240-0.

## Graphical abstract

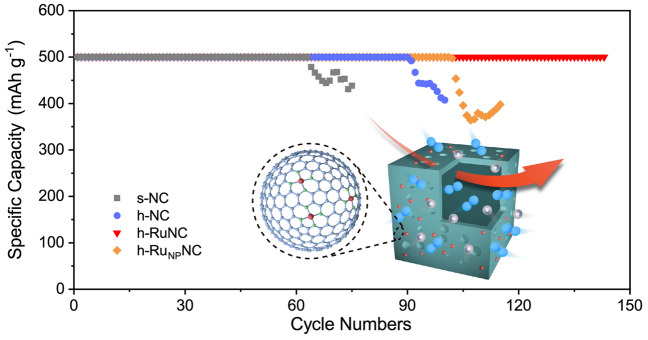

## Introduction

Lithium–oxygen (Li–O_2_) batteries, due to their ultra-high theoretical energy density, have shown enormous application potential in facilitating energy transformation in the future and achieving large-scale energy storage [1–5]. However, due to the insolubility and insulation of the discharge product lithium peroxide (Li_2_O_2_), the redox kinetics in the battery is slow, exhibiting a large overpotential in the charge and discharge process [6–10]. Meanwhile, the insolubility of the Li_2_O_2_ and the low diffusion efficiency of catalytic molecules, such as Li^+^ and O_2_, consistently result in inadequate stability [11–16]. The aforementioned two challenges pose significant obstacles to the commercialization progress of Li–O_2_ batteries [17–19]. Based on this, there is a pressing need to design effective air cathode catalysts that not only possess an adequate number of highly active sites but also demonstrate accessible active sites and enable the rapid transfer of reactant molecules [20–26].

The advantages of single-atom catalysts such as ultra-high atomic utilization and unsaturated coordination of active sites endow them with high catalytic activity, demonstrating significant catalytic advantages in many fields such as fuel cells, water splitting and organic small molecule catalysis [27–35]. In Li–O_2_ battery, some single-atom catalysts have been studied in recent years, and good results have been achieved in the improving of battery performance [36–40]. Among them, the atomically dispersed metal–nitrogen–carbon (M–N–C) catalyst prepared with metal organic frameworks (MOFs), especially zeolite imidazolate frameworks (ZIFs) as the precursor, have been widely used due to their advantages of rich micropores and high specific surface area, but the solid structure reduces the mass activity of catalysts and the accessibility of the internal active site [41, 42]. And the microporous structure also limits the diffusion of catalytic molecules. To address these issues, fortunately, the open hollow structure has the capability to effectively expose a greater number of active sites [43–45]. For example, Zhuang et al. developed a hollow RuIrO_*x*_ nano-netcages with three-dimensional structure to achieve efficient pH-universal for overall water splitting [46]. Han et al. [47] obtained a hollow, mesoporous, atomically dispersed M–N–C catalysts with enhanced catalytic diffusion, achieving efficient conversion of organic macromolecules. In addition, the hollow structure can also show greater advantages in Li–O_2_ battery, providing sufficient storage space for the generation of Li_2_O_2_ and improving the mass transfer efficiency of Li^+^ and O_2_ [48]. Therefore, based on these considerations, it is reasonable to expect that the design of atomically dispersed catalysts with open hollow structure for Li–O_2_ battery will improve the reaction kinetics effectively and achieve the improvement of battery cycling stability.

Here, we reported a N-doped carbon anchored atomically dispersed Ru sites catalyst with open hollow structure. Using ZIF-8 as the precursor, the internal dead volume was holed out by chemical etching to obtain a cavity of about 200 nm, and the surface was rich in mesopores, which improved the diffusion efficiency of catalytic molecules and realized the rapid mass transfer of electrolyte and oxygen. X-ray absorption spectroscopy (XAS) and aberration-corrected high-angle annular dark-field scanning transmission electron microscopy (AC HAADF-STEM) confirmed the existence of abundant atomically dispersed Ru sites in the catalyst, providing a large number of oxygen adsorption and Li_2_O_2_ nucleation sites, while the open hollow structure further improved the dispersity and accessibility of the Ru active sites. With the excellent activity from atomically dispersed Ru sites and the enhanced diffusion from open hollow structure, the generation and decomposition efficiency of Li_2_O_2_ in Li–O_2_ battery was improved, a large discharge specific capacity and cycling stability of nearly 150 cycles were obtained. This provides more ideas for improving catalyst activity.

## Experimental Section

### Preparation of Materials

#### Preparations of ZIF-8

In a typical synthesis [46], 5.6752 g 2-methylimidazole was dispersed in 87.2 mL deionized water containing 17.5 mg hexadecyl trimethyl ammonium bromide (CTAB) labeled as solution A. 0.3626 g Zn(NO_3_)_2_·6H_2_O was dispersed in 12.5 mL deionized water labeled as solution B. Under stirring, solution B was injected into solution A, and the mixture was stirred for 5 min and then left at rest at 28 °C for 3 h. The products were obtained by centerfugation, washed several times with deionized water, and dried.

#### Preparations of h-ZIF-8

The ZIF-8 obtained above was dispersed in 20 mL ethanol. 350 mg tannic acid (TA) was dissolved in 300 mL of deionized water and ethanol (1:1 volume of the two mixed solutions). Then, stirring at room temperature for 10 min.

#### Preparations of s-NC and h-NC

s-NC was obtained by annealing the ZIF-8 at 900 °C for 2 h in the atmosphere of nitrogen. h-NC was obtained by annealing the H-ZIF-8 at 900 °C for 2 h in the atmosphere of nitrogen.

#### Preparations of h-RuNC, s-RuNC and h-Ru_NP_NC

h-NC powder was dispersed in 20 mL of isopropanol, and then Ru solution with 2% mass fraction was dissolved in 5 mL of isopropanol. The Ru solution was added drop by drop to the dispersion of h-NC under untrasound and continued for 30 min, follwed by stirring for 12 h. Centrifuge the precipitate, wash with isopropanol for 3 times, and dry. Then, the powder annealed at 500 °C for 2 h in the atmosphere of nitrogen, and obtained h-RuNC. The synthesis of s-RuNC was similar to that of h-RuNC, except that h-NC was replaced by s-NC. The synthesis of h-Ru_NP_NC was similar to that of h-RuNC, only 5% Ru solution was added.

### Characterizations

The crystal phases were identified using powder X-ray diffractometer (XRD) on a Phillips X’pertProMPD with Cu Kα radiation. Catalyst morphology was obtained using scanning electron microscope (SEM) on a S-4800 Hitachi. Transmission electron microscopy (TEM) images were observed on a Hitachi H-800 TEM working at 100 kV. High angle annular dark field scanning TEM (HAADF–STEM) and corresponding energy dispersive X-ray spectroscopy (EDS) images were performed on a Titan 80–300 STEM operated at 300 kV. N_2_ adsorption–desorption measurements were carried out at 77 K, the Brunauer–Emmett–Teller (BET) surface area was calculated using experimental points at a relative pressure of *P*/*P*_o_ = 0.05–1.00. Elemental analysis of Ru in the samples was performed on a Thermo IRIS Intrepid II inductively coupled plasma-optical emission spectrometry (ICP-OES). X-ray photoelectron spectroscopy (XPS) analyses were conducted on ESCALAB 250 Xi X-ray photoelectron spectrometer with Al Kα radiation. Raman spectra were obtained on a microscopic confocal Raman spectrometer (LabRAM Aramis, Horiba Jobin Yvon) with a 532 nm laser. The X-ray absorption fine structure (XAFS) spectra were collected at 1W1B station in BSRF (Beijing Synchrotron Radiation Facility, P. R. China) operated at 2.5 GeV with a maximum current of 250 mA.

### Battery Assembly and Performance

The air electrode was composed of 90% as-synthesized catalyst and 10% polyvinylidene fuoride (PVDF). The total mass of the electrodes is 0.6–0.7 mg. The assembled button battery model was CR2032, and the battery consisted of a lithium foil, glass fiber filter, air electrode and electrolyte (1 M lithium bis(trifluoromethylsulfonyl)imide, LiTFSI dissolved in a tetra(ethylene) glycol dimethyl ether, TEGDME), assembled in a glove box filled with argon gas. The galvanostatic discharge–charge curve was tested on a Neware battery test system at the voltage range of 2.0–4.5 V.

## Results and Discussion

### Morphological Characterization of h-RuNC

**Fig. 1:**
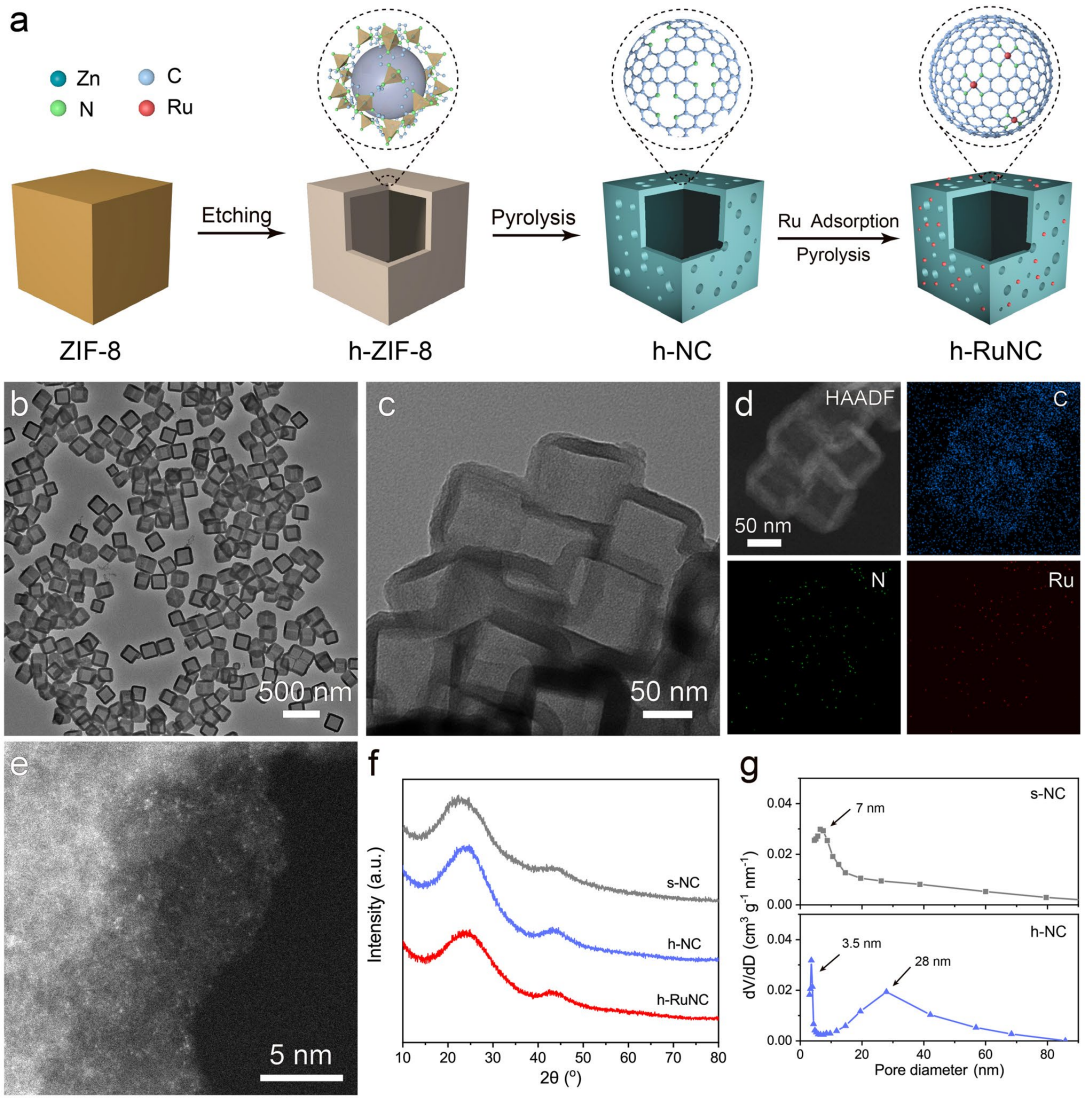
**a** Schematic illustration of the fabrication of h-RuNC. **b** TEM image of h-ZIF-8. **c** TEM image of h-RuNC. **d** HAADF-STEM and corresponding elemental mapping images of h-RuNC. **e** AC HAADF-STEM image of h-RuNC. **f** XRD patterns of catalysts. **g** Pore size distribution of s-NC and h-NC

Figure 1a shows a schematic diagram of the design synthesis process of catalysts. We first synthesized ZIF-8 in uniform size (Fig. S1), and then used ZIF-8 as a precursor to hollow out the cube through the action of tannic acid (TA) as surface functionalized and etching agent to obtain a hollow structure, the internal cavity diameter was about 150–200 nm (Figs. 1b and S2), denoted as h-ZIF-8. Then, after heat treatment at 900 °C in nitrogen atmosphere for 2 h, hollow nitrogen-doped carbon nanocages were obtained, denoted as h-NC (Fig. S3). Ru sites were loaded on the surface of the hollow nanocages through electrostatic adsorption, denoted as h-RuNC (Figs. 1c and S4). It can be observed by EDS images that Ru, C and N are uniformly distributed on the three-dimensional framework in h-RuNC (Fig. 1d). The dispersion state of Ru in h-RuNC was further observed by AC HAADF-STEM, and it is found that Ru existed in an atomically dispersed state on h-RuNC, which is the highlight position in the figure (Figs. 1e and S5). The above results indicate that we have successfully synthesized nitrogen-doped carbon anchored atomically dispersed Ru cathode catalyst with open hollow structure. XRD patterns also further indicates that h-RuNC is amorphous carbon and there is no crystalline phase of Ru (Fig. 1f). The specific surface area of h-NC is 940 m^2^ g^–1^ (Fig. S6), and the surface has a large number of porous structures, and a bimodal pore size distribution of 3.5 and 28 nm, respectively (Fig. 1g). In addition, we compared the solid cube that had not been hollowed out by etching agent and denoted as s-NC. The specific surface area of s-NC is 668 m^2^ g^–1^, indicating that after etching with an etchant, h-NC has a higher specific surface area and generates more abundant pore structures on its surface.

**Fig. 2:**
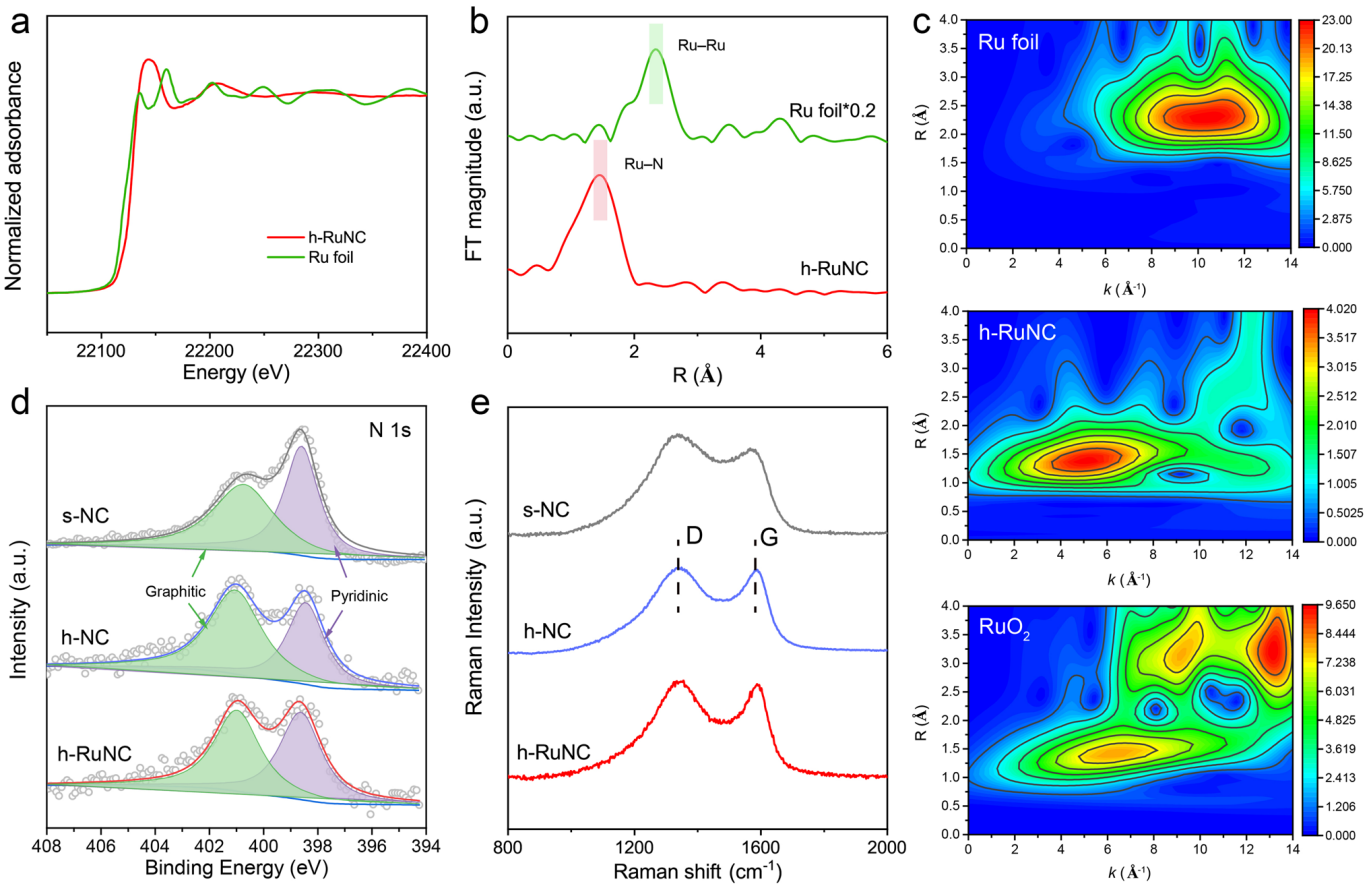
**a** Normalized XANES spectra of Ru K-edge. **b** Fourier-transform EXAFS spectra of R space. **c** Wavelet transform of Ru foil, h-RuNC and RuO_2_. **d** XPS N 1*s* spectra of s-NC, h-NC and h-RuNC. **e** Raman spectra of s-NC, h-NC and h-RuNC

We further studied the electronic structure and coordination environment of Ru atoms in h-RuNC by XAFS. The X-ray absorption near edge structure (XANES) spectra of Ru k-edge show that Ru atoms are positively charged in h-RuNC (Fig. 2a). The extended edge X-ray absorption fine structure (EXAFS) spectrum of h-RuNC has a main peak at 1.4 Å, corresponding to the first coordination shell of Ru–N [29], and no obvious Ru–Ru peak, indicating that Ru is atomically disperse anchored on the carrier (Fig. 2b). Then, the wavelet transform (WT) analysis of EXAFS signal of Ru K-edge (Fig. 2c), it can be observed that the maximum intensity at 2.3 Å^–1^ belongs to Ru–Ru in the spectrum of Ru foil [49], the maximum intensity at 1.4 Å^–1^ belongs to Ru–O in the spectrum of RuO_2_, and the maximum WT contour of h-RuNC assigned to Ru–N contribution, further confirming the atomic dispersion state of Ru. EXAFS fitting reveals the coordination environment of Ru in h-RuNC, it could be deduced that each Ru atom is coordinated with four N atoms, forming the RuN_4_ site (Fig. S7 and Table S1). Next, we characterized the surface electronic structure of these catalysts by XPS, and Ru, N, C and O elements have obvious signals (Fig. S8). In the N 1*s* spectra (Fig. 2d), two well-separated peaks are shown, corresponding to graphite N (401.1 eV) and pyridine N (398.5 eV), respectively [50]. Surprisingly, we find that the ratio of graphite *N*/pyridine *N* in s-NC is 1.04. However, after modification with etchants, the ratio of graphite *N*/pyridine *N* in h-NC is 1.43, the proportion of graphite *N* in h-NC is significantly increased, becoming the dominant type of N species. Therefore, after modification with etchants, not only does it remove the dead volume inside the cube, but it also regulates the type of *N* species. After the further introduction of Ru, a high proportion of graphite *N* was still maintained. As shown in Fig. S9 of Ru 3*p* spectrum, the oxidation state of Ru in h-RuNC is positively charged. The C 1*s* spectra show that the carbon structure does not change significantly during etching agent treatment and Ru loading (Fig. S10). In addition, Raman spectroscopy shows that the proportion of D and G peaks in h-NC and h-RuNC decreased after etching agent treatment, indicating a better graphitic structure and higher conductivity, which is conducive to the electron transfer for charging and discharging processes (Fig. 2e).

In order to further understand the catalytic active center, we loaded a higher content of Ru on the surface of h-NC, denoted as h-Ru_NP_NC (Fig. S11). The introduction of a high load of Ru did not significantly affect the structure of h-NC, and the crystalline phase of Ru was not shown in XRD (Fig. S12). Therefore, we further observed h-Ru_NP_NC through HAADF-STEM (Fig. S13), and found that the content of Ru on its surface increased. Ru, C and N were uniformly distributed on the three-dimensional framework, and Ru particles were observed in h-Ru_NP_NC. The content of Ru in h-RuNC and h-Ru_NP_NC was 1.24% and 2.70%, respectively, by ICP-OES analysis (Table S2).

### Electrocatalytic Performance

**Fig. 3:**
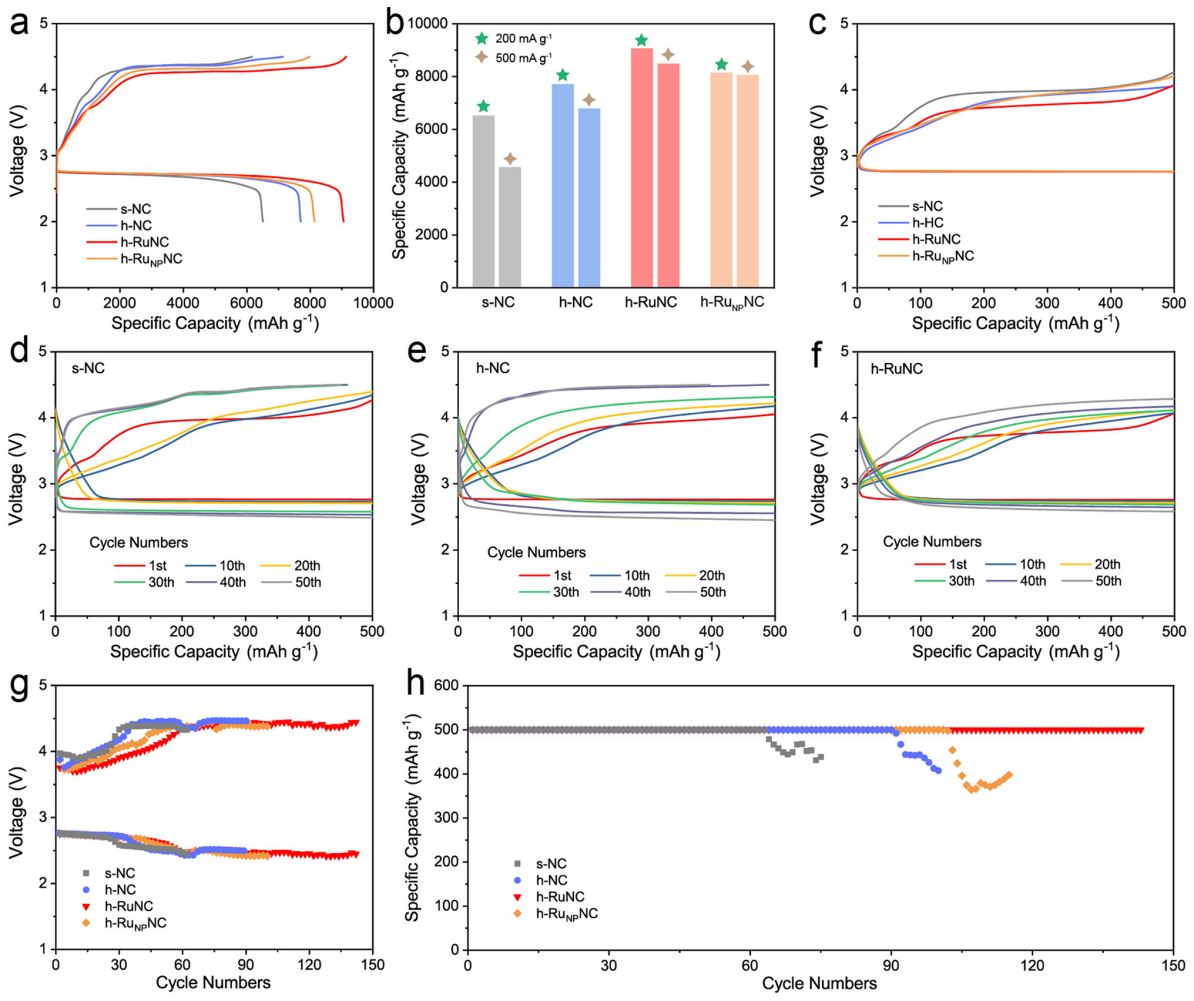
**a** Full discharge/charge curves of as-synthesized catalysts at the current density of 200 mA g^–1^. **b** Comparison of discharge capacities of different catalysts at different current densities. **c** First discharge/charge curves of as-synthesized catalysts. Discharge/charge curves of **d** s-NC, **e** h-NC and **f** h-RuNC. **g** Plots of medium voltage against cycling number. **h** Cycling performances of the as-synthesized catalysts

This series of catalysts were applied to Li–O_2_ battery to investigate the effect of catalyst structure and active site on the performance of batteries. The full discharge in Fig. 3a shows that after modification by etching agent, the discharge capacity of h-NC is significantly improved, which is the result that we can foresee, because the cavity in h-NC can provide more storage space for discharge products Li_2_O_2_, and the discharge specific capacity of Li–O_2_ battery is achieved by the reaction of lithium ion and oxygen to generate Li_2_O_2_. The more Li_2_O_2_ can be generated, the higher the discharge capacity. In addition, h-NC greatly increases the surface quantity and unit mass activity of the catalyst due to the removal of internal dead volume. h-RuNC obtained more active sites due to the introduction of atomically dispersed Ru, and the discharge specific capacity was further increased. However, the discharge specific capacity of h-Ru_NP_NC decreased after the formation of Ru particles. Then the current density was increased to 500 mA g^−1^, h-RuNC still shows a high discharge capacity (Fig. 3b). When the current density is 200 mA g^–1^ and the limited capacity is 500 mAh g^–1^, the first charge and discharge curve show that h-RuNC has the lowest charge and discharge overpotential, showing good reversibitliy (Fig. 3c). Subsequently, we investigated the long-term stable cycling of the battery. By observing the turn-by-turn charge–discharge curves (Fig. 3d-f), we found that the overpotential of s-NC increased rapidly, h-NC could delay the growth of overpotential to a certain extent, and h-RuNC could effectively control the groth of overpotential. This indicates that the formation of open hollow structure and mesopores can accelerate the diffusion efficienty of the catalytic molecules and improve the reaction rate of the discharge process, while h-RuNC further introduces atomically dispersed Ru sites with high catalytic activity, effectively improving the catalytic decomposition ability of Li_2_O_2_. The final cycling results showed that h-RuNC could stably cycle for nearly 150 times, while s-NC cycles for 60 times, h-NC cycles for 90 times, and h-Ru_NP_NC cycles for less than 100 times (Fig. 3g, h).

In addition, we tested the cyclic voltammetry curves of the catalyst before and after the introduction of atomically dispersed Ru sites (Fig. S14), and found that the peak current intensity of h-RuNC invreased significantly in oxygen reduction reaction (ORR) and oxygen evolution reaction (OER), indicating that the introduction of atomically dispersed Ru sites had significantly improved the catalytic activity of the catalyst. We further validated the advantages of open hollow structure by loading Ru with the same content as h-RuNC on solid s-NC, denoted as s-RuNC (Fig. S15). Through XAFS, it could be seen that Ru also exists in atomically dispersed state in s-RuNC (Fig. S16), which is consistent with the XRD result in Fig. S15b. By observing the cycling curves of battery based on s-RuNC, we find that the overpotential of s-RuNC is larger than that of h-RuNC, and the number of cycles also decreased, which can cycle for 100 times (Fig. S17). This shows that the open hollow structure has an impact on the diffusion of catalytic molecular, which in turn affects the cycling of the battery. The electrochemical activity of s-RuNC has also been significantly improved compared to s-NC, indicating the importance of atomically dispersed Ru for the improvement of activity. We also observed the morphology of h-RuNC after 150 cycles. The three-dimensional strucutre of the catalyst remained intact, with the majority Ru still existing in an atomically dispersed state, while a small amount of Ru was also agglomerated into clusters (Fig. S18).

### Morphology of Discharge Product

**Fig. 4:**
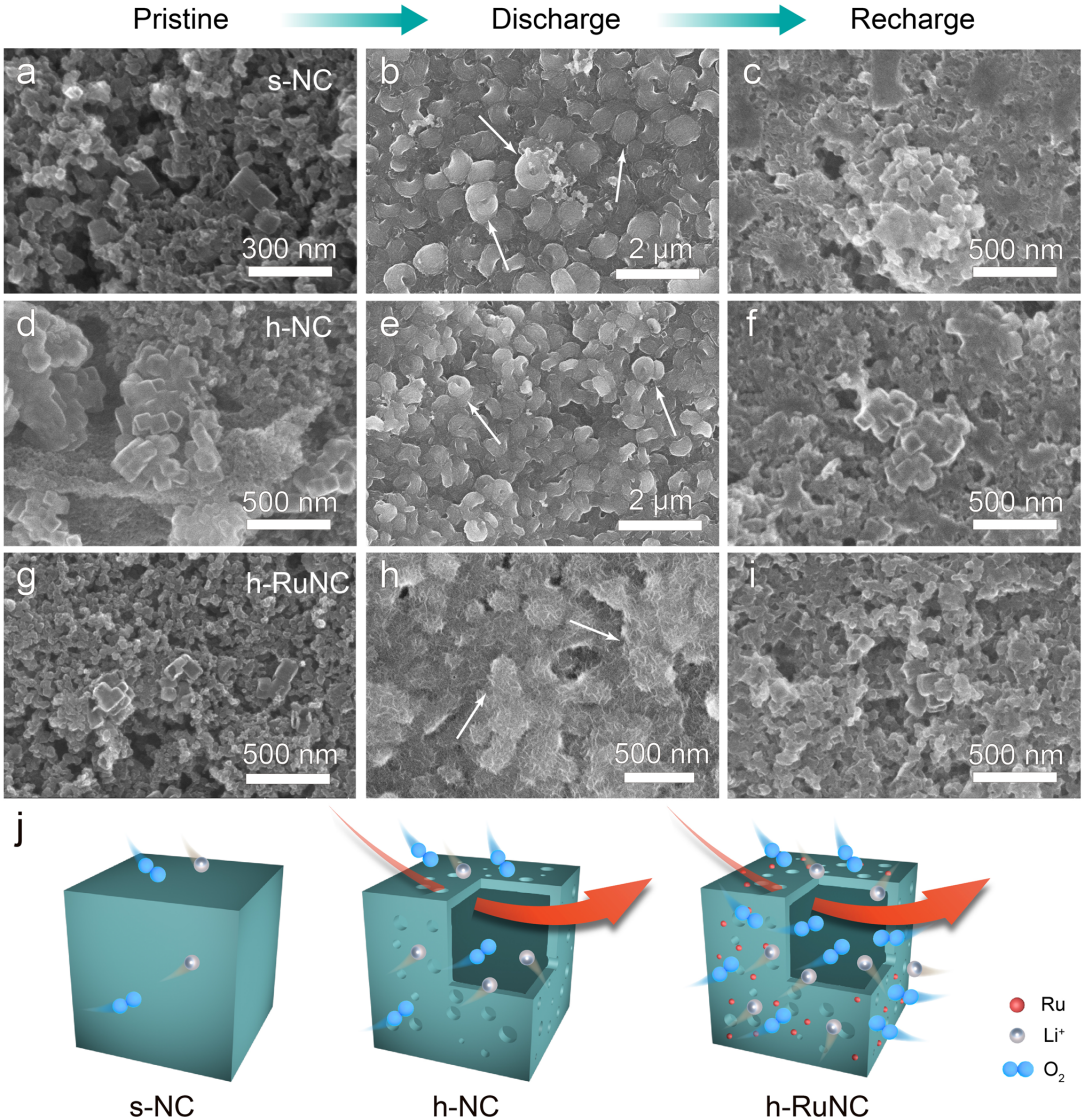
SEM images of the cathodes after discharge and recharge **a-c** s-NC. **d-e** h-NC. **g-h** h-RuNC at different states. **a, d, g** pristine, **b, e, h** after full discharge, **c, f, i** after recharge.** j** Schematic diagram of catalytic activity enhancement from s-NC to h-NC to h-RuNC

Furthermore, we observed the morphology and phase of the discharge products. Firstly, the discharge products catalyzed by these catalysts were characterized by XRD (Fig. S19), and it was found that the discharge products were Li_2_O_2_. Subsequently, we observed the morphology of Li_2_O_2_ and conducted ex-situ SEM analysis on the surface of the electrodes with different Li_2_O_2_ (Figs. 4 and S20). Firstly, the catalyst, KB and binder can be observed on the surface of pristine electrode (Fig. 4a, d, g). After the discharge process, the discharge products can be clearly observed on the surface of these catalyst electrodes. With the extension of the discharge depth, the discharge products further grow. The discharge products catalyzed by s-NC and h-NC show a typical toroidal shape (Fig. 4b, e), while the discharge products catalyzed by h-RuNC show a film shape with higher dispersion (Fig. 4h). Therefore, after subsequent recharging, the discharge products generated by h-RuNC are more easily and efficiently decomposed (Fig. 4c, f, i) [51, 52]. It is precisely due to the efficient decomposition of discharge products that the cycling stability of battery has been significantly improved. We further analyzed the electronic structures of the discahrge products on the electrode surfaces after the discharge process of these catalsyts using XPS. As shown in Fig. S21, in addition to the main discharge product Li_2_O_2_, a small amount of Li_2_CO_3_ was also produced. However, it is obvious that among several catalysts, the proportion of byproduct Li_2_CO_3_ in the discharge product of h-RuNC is the least. Thus, the h-RuNC catalyst can decompose efficiently the discharg product and maintain a long-term stable cycle of the battery.

On the whole, there are a large number of atomically dispersed Ru sites on the surface of h-RuNC, which can adsorb a large amount of oxygen during the discharge process, providing more nucleation sites for Li_2_O_2_, improving the discharge specific capacity of the battery, and guiding the discharge products to grow into a film shape with higher dispersion through surface growth mode; Subsequently, during the charging process, the discharge products are more easily decomposed, resulting in a low charging overpotential for the battery, improving the performance of the battery. Conversely, the discharge products of other catalysts continuously migrate on the catalyst surface and grow into large toroidal shape, resulting in poor decomposition efficiency during charging.

## Conclusions

In summary, we obtained N-doped carbon anchored atomically dispersed Ru with open hollow structure as cathode catalyst by etching strategy. By creating open hollow structures and regulating the dispersion state of Ru on N-doped carbon, h-RuNC exhibited superior electrochemical performance compared to Ru particle (h-Ru_NP_NC) and solid structure (s-RuNC) in Li–O_2_ battery, and could cycling stably for nearly 150 cycles. The open hollow structure can improve the diffusion efficiency of catalytic molecules, which is very suitable for the mass transfer process of Li^+^ and O_2_ in Li–O_2_ battery, and improve the accessibility of active sites of the catalyst. And through the analysis results of XPS, we found that after the etching agent treatment, the type of N in the carrier has a regulatory effect, which can increase the proportion of graphite N. The large number of atomically dispersed Ru sites also improved the formation and decomposition efficiency of discharge products effectively, and the battery also achieved a discharge specific capacity of up to 1000 mAh g^–1^. Thanks to the dual optimization of structure and active site (Fig. 4j), h-RuNC has excellent performance in Li–O_2_ battery, which provides an important idea for optimizing the application of high-performance atomically dispersed M–N–C catalysts in Li–O_2_ battery.

## Supplementary Information

Below is the link to the electronic supplementary material.

Supplementary file1 (PDF 1847 kb)

## References

[1] J. Xu, J. Ma, Q. Fan, S. Guo, S. Dou, Recent progress in the design of advanced cathode materials and battery models for high-performance lithium-X (X = O_2_, S, Se, Te, I_2_, Br_2_) Batteries. Adv. Mater. **29**(28), 1606454 (2017). 10.1002/adma.201606454 10.1002/adma.201606454 28488763

[2] A.S. Arico, P. Bruce, B. Scrosati, J.M. Tarascon, W. Van Schalkwijk, Nanostructured materials for advanced energy conversion and storage devices. Nat. Mater. **4**(5), 366–377 (2005). 10.1038/nmat1368 15867920 10.1038/nmat1368

[3] K. Noah, L. Felix, M.K. Daniel, Energy storage deployment and innovation for the clean energy transition. Nat. Energy **2**, 17125 (2017). 10.1038/nenergy.2017.125

[4] Y.Y. Birdja, E. Pérez-Gallent, M.C. Figueiredo, A.J. Göttle, F. Calle-Vallejo et al., Advances and challenges in understanding the electrocatalytic conversion of carbon dioxide to fuels. Nat. Energy **4**(9), 732–745 (2019). 10.1038/s41560-019-0450-y

[5] W.J. Kwak, Rosy, D. Sharon, C. Xia, H. Kim et al., Lithium–oxygen batteries and related systems: potential, status, and future. Chem. Rev. **120**, 6626–6683 (2020). 10.1021/acs.chemrev.9b00609 32134255 10.1021/acs.chemrev.9b00609

[6] D. Geng, N. Ding, T.S.A. Hor, S.W. Chien, Z. Liu et al., From lithium–oxygen to lithium-air batteries: challenges and opportunities. Adv. Energy Mater. **6**(9), 1502164 (2016). 10.1002/aenm.201502164

[7] L. Ma, T. Yu, E. Tzoganakis, K. Amine, T. Wu et al., Fundamental understanding and material challenges in rechargeable nonaqueous Li–O_2_ batteries: recent progress and perspective. Adv. Energy Mater. **8**(22), 1800348 (2018). 10.1002/aenm.201800348

[8] Q. Dong, X. Yao, Y. Zhao, M. Qi, X. Zhang et al., Cathodically stable Li–O_2_ battery operations using water-in-salt electrolyte. Chem **4**(6), 1345–1358 (2018). 10.1016/j.chempr.2018.02.015

[9] G. Li, N. Li, S. Peng, B. He, J. Wang et al., Highly efficient Nb_2_C MXene cathode catalyst with uniform O-terminated surface for lithium–oxygen batteries. Adv. Energy Mater. (2020). 10.1002/aenm.202002721

[10] M. Yuan, R. Wang, W. Fu, L. Lin, Z. Sun et al., Ultrathin two-dimensional metal organic framework nanosheets with the inherent open active sites as electrocatalysts in aprotic Li–O_2_ batteries. ACS Appl. Mater. Interfaces **11**(12), 11403–11413 (2019). 10.1021/acsami.8b21808 30816695 10.1021/acsami.8b21808

[11] H. Wang, X. Wang, M. Li, L. Zheng, D. Guan et al., Porous materials applied in nonaqueous Li–O_2_ batteries: status and perspectives. Adv. Mater. **32**(44), e2002559 (2020). 10.1002/adma.202002559 32715511 10.1002/adma.202002559

[12] C. Li, Z. Guo, Y. Pang, Y. Sun, X. Su et al., Three-dimensional ordered macroporous FePO_4_ as high-efficiency catalyst for rechargeable Li–O_2_ batteries. ACS Appl. Mater. Interfaces **8**(46), 31638–31645 (2016). 10.1021/acsami.6b10115 27797471 10.1021/acsami.6b10115

[13] Z. Guo, D. Zhou, X. Dong, Z. Qiu, Y. Wang et al., Ordered hierarchical mesoporous/macroporous carbon: a high-performance catalyst for rechargeable Li–O_2_ batteries. Adv. Mater. **25**(39), 5668–5672 (2013). 10.1002/adma.201302459 23913835 10.1002/adma.201302459

[14] S. Huiyu, X. Shaomao, L. Yiju, D. Jiaqi, G. Amy et al., Hierarchically porous, ultrathick, “breathable” wood-derived cathode for lithium–oxygen batteries. Adv. Energy Mater. **8**, 1701203 (2017). 10.1002/aenm.201701203

[15] J. Kang, J. Kim, S. Lee, S. Wi, C. Kim et al., Breathable carbon-free electrode: Black TiO_2_ with hierarchically ordered porous structure for stable Li–O_2_ battery. Adv. Energy Mater. **7**(19), 1700814 (2017). 10.1002/aenm.201700814

[16] X.X. Wang, D.H. Guan, C.L. Miao, D.C. Kong, L.J. Zheng et al., Metal-organic framework-based mixed conductors achieve highly stable photo-assisted solid-state lithium-oxygen batteries. J. Am. Chem. Soc. **145**(10), 5718–5729 (2023). 10.1021/jacs.2c11839 36880105 10.1021/jacs.2c11839

[17] L.-J. Zheng, P. Bai, W.-F. Yan, F. Li, X.-X. Wang et al., In situ construction of glass-fiber-directed zeolite microtube woven separator for ultra-high-capacity lithium–oxygen batteries. Matter **6**, 1–16 (2022). 10.1016/j.matt.2022.10.013

[18] R. Rojaee, R. Shahbazian-Yassar, Two dimensional materials to address the Li-based battery challenges. ACS Nano **14**(3), 2628–2658 (2020). 10.1021/acsnano.9b08396 32083832 10.1021/acsnano.9b08396

[19] Y. Zhang, S. Zhang, J. Ma, X. Chen, C. Nan et al., Single-atom-mediated spinel octahedral structures for elevated performances of Li–oxygen batteries. Angew. Chem. Int. Ed. **62**, e202218926 (2023). 10.1002/ange.202218926 10.1002/anie.202218926 36786069

[20] P. Zhang, Y. Zhao, X. Zhang, Functional and stability orientation synthesis of materials and structures in aprotic Li–O_2_ batteries. Chem. Soc. Rev. **47**(8), 2921–3004 (2018). 10.1039/C8CS00009C 29577147 10.1039/c8cs00009c

[21] K.X. Wang, Q.C. Zhu, J.S. Chen, Strategies toward high-performance cathode materials for lithium-oxygen batteries. Small **14**(27), e1800078 (2018). 10.1002/smll.201800078 29750439 10.1002/smll.201800078

[22] X. Wang, Q. Dong, H. Qiao, Z. Huang, M.T. Saray et al., Continuous synthesis of hollow high-entropy nanoparticles for energy and catalysis applications. Adv. Mater. **32**, e2002853 (2020). 10.1002/adma.202002853 33020998 10.1002/adma.202002853

[23] G. Tian, L. Ren, H. Xu, T. Zeng, X. Wang et al., Metal sulfide heterojunction with tunable interfacial electronic structure as an efficient catalyst for lithium-oxygen batteries. Sci. China Mater. **66**(4), 1341–1351 (2023). 10.1007/s40843-022-2253-y

[24] Y. Zhou, K. Yin, Q. Gu, L. Tao, Y. Li et al., Lewis-acidic ptir multipods enable high-performance Li–O_2_ batteries. Angew. Chem. Int. Ed. **60**(51), 26592–26598 (2021). 10.1002/anie.202114067 10.1002/anie.202114067 34719865

[25] T. Jin, J. Nie, M. Dong, B. Chen, J. Nie et al., 3D Interconnected honeycomb-like multifunctional catalyst for Zn-air batteries. Nano-Micro Lett. **15**, 26 (2022). 10.1007/s40820-022-00959-6 10.1007/s40820-022-00959-6 PMC9805485 36586003

[26] H. Chen, Y. Ye, X. Chen, L. Zhang, G. Liu et al., N-doped porous carbon nanofibers inlaid with hollow Co_3_O_4_ nanoparticles as an efficient bifunctional catalyst for rechargeable Li–O_2_ batteries. Chin. J. Catal. **43**(6), 1511–1519 (2022). 10.1016/S1872-2067(21)64017-2

[27] J. Li, M. Chen, D.A. Cullen, S. Hwang, M. Wang et al., Atomically dispersed manganese catalysts for oxygen reduction in proton-exchange membrane fuel cells. Nat. Catal. **1**(12), 935–945 (2018). 10.1038/s41929-018-0164-8

[28] X. Wang, N. Fu, J.C. Liu, K. Yu, Z. Li et al., Atomic replacement of PtNi nanoalloys within Zn-ZIF-8 for the Fabrication of a Multisite CO_2_ reduction electrocatalyst. J. Am. Chem. Soc. **144**(50), 23223–23229 (2022). 10.1021/jacs.2c11497 36490370 10.1021/jacs.2c11497

[29] K. Sun, X. Wu, Z. Zhuang, L. Liu, J. Fang et al., Interfacial water engineering boosts neutral water reduction. Nat. Commun. **13**, 6260 (2022). 10.1038/s41467-022-33984-5 36271080 10.1038/s41467-022-33984-5 PMC9587018

[30] C. Liu, Y. Wu, K. Sun, J. Fang, A. Huang et al., Constructing FeN_4_/graphitic nitrogen atomic interface for high-efficiency electrochemical CO_2_ reduction over a broad potential window. Chem **7**(5), 1297–1307 (2021). 10.1016/j.chempr.2021.02.001

[31] W.H. Li, J. Yang, H. Jing, J. Zhang, Y. Wang et al., Creating high regioselectivity by electronic metal-support interaction of a single-atomic-site catalyst. J. Am. Chem. Soc. **143**(37), 15453–15461 (2021). 10.1021/jacs.1c08088 34506145 10.1021/jacs.1c08088

[32] Z. Chen, A. Huang, K. Yu, T. Cui, Z. Zhuang et al., Fe_1_N_4_–O_1_ site with axial Fe–O coordination for highly selective CO_2_ reduction over a wide potential range. Energy Environ. Sci. **14**(6), 3430–3437 (2021). 10.1039/d1ee00569c

[33] Y. Chen, S. Ji, C. Chen, Q. Peng, D. Wang et al., Single-atom catalysts: synthetic strategies and electrochemical applications. Joule **2**(7), 1242–1264 (2018). 10.1016/j.joule.2018.06.019

[34] S. Ji, Y. Chen, Q. Fu, Y. Chen, J. Dong et al., Confined pyrolysis within metal-organic frameworks to form uniform Ru_3_ clusters for efficient oxidation of alcohols. J. Am. Chem. Soc. **139**(29), 9795–9798 (2017). 10.1021/jacs.7b05018 28696113 10.1021/jacs.7b05018

[35] Z. Pu, I.S. Amiinu, R. Cheng, P. Wang, C. Zhang et al., Single-atom catalysts for electrochemical hydrogen evolution reaction: recent advances and future perspectives. Nano-Micro Lett. **12**, 21 (2020). 10.1007/s40820-019-0349-y 10.1007/s40820-019-0349-y PMC7770676 34138058

[36] Q. Lv, Z. Zhu, Y. Ni, B. Wen, Z. Jiang et al., Atomic ruthenium-riveted metal-organic framework with tunable d-band modulates oxygen redox for lithium-oxygen batteries. J. Am. Chem. Soc. **144**(50), 23239–23246 (2022). 10.1021/jacs.2c11676 36474358 10.1021/jacs.2c11676

[37] D. Zhao, P. Wang, H. Di, P. Zhang, X. Hui et al., Single semi-metallic selenium atoms on Ti_3_C_2_ MXene nanosheets as excellent cathode for lithium–oxygen batteries. Adv. Funct. Mater. **31**(29), 2010544 (2021). 10.1002/adfm.202010544

[38] P. Wang, Y. Ren, R. Wang, P. Zhang, M. Ding et al., Atomically dispersed cobalt catalyst anchored on nitrogen-doped carbon nanosheets for lithium-oxygen batteries. Nat. Commun. **11**(1), 1576 (2020). 10.1038/s41467-020-15416-4 32221290 10.1038/s41467-020-15416-4 PMC7101366

[39] L.N. Song, W. Zhang, Y. Wang, X. Ge, L.C. Zou et al., Tuning lithium-peroxide formation and decomposition routes with single-atom catalysts for lithium-oxygen batteries. Nat. Commun. **11**(1), 2191 (2020). 10.1038/s41467-020-15712-z 32366827 10.1038/s41467-020-15712-z PMC7198606

[40] X. Hu, G. Luo, Q. Zhao, D. Wu, T. Yang et al., Ru single atoms on N-doped carbon by spatial confinement and ionic substitution strategies for high-performance Li–O_2_ batteries. J. Am. Chem. Soc. **142**(39), 16776–16786 (2020). 10.1021/jacs.0c07317 32876448 10.1021/jacs.0c07317

[41] N. Li, Z. Chang, M. Zhong, Z.-X. Fu, J. Luo et al., Functionalizing MOF with redox-active tetrazine moiety for improving the performance as cathode of Li–O_2_ batteries. CCS Chem. **3**(3), 1297–1305 (2021). 10.31635/ccschem.020.202000284

[42] Y. Pan, K. Sun, S. Liu, X. Cao, K. Wu et al., Core-shell ZIF-8@ZIF-67-derived CoP nanoparticle-embedded N-doped carbon nanotube hollow polyhedron for efficient overall water splitting. J. Am. Chem. Soc. **140**(7), 2610–2618 (2018). 10.1021/jacs.7b12420 29341596 10.1021/jacs.7b12420

[43] C. Chen, Y. Kang, Z. Huo, Z. Zhu, W. Huang et al., Highly crystalline multimetallic nanoframes with three-dimensional electrocatalytic surfaces. Science **343**, 1339–1343 (2014). 10.1126/science.1249061 24578531 10.1126/science.1249061

[44] Y. Xia, X. Yang, Toward cost-effective and sustainable use of precious metals in heterogeneous catalysts. Acc. Chem. Res. **50**(3), 450–454 (2017). 10.1021/acs.accounts.6b00469 28945412 10.1021/acs.accounts.6b00469

[45] M. Zhao, X. Wang, X. Yang, K.D. Gilroy, D. Qin et al., Hollow metal nanocrystals with ultrathin, porous walls and well-controlled surface structures. Adv. Mater. **30**(48), e1801956 (2018). 10.1002/adma.201801956 29984540 10.1002/adma.201801956

[46] Z. Zhuang, Y. Wang, C.-Q. Xu, S. Liu, C. Chen et al., Three-dimensional open nano-netcage electrocatalysts for efficient pH-universal overall water splitting. Nat. Commun. **10**, 4875 (2019). 10.1038/s41467-019-12885-0 31653856 10.1038/s41467-019-12885-0 PMC6814841

[47] X. Han, T. Zhang, X. Wang, Z. Zhang, Y. Li et al., Hollow mesoporous atomically dispersed metal-nitrogen-carbon catalysts with enhanced diffusion for catalysis involving larger molecules. Nat. Commun. **13**, 2900 (2022). 10.1038/s41467-022-30520-3 35610219 10.1038/s41467-022-30520-3 PMC9130124

[48] Y. Zhang, S. Zhang, M. Yuan, Y. Li, R. Liu et al., Optimal geometrical configuration and oxidation state of cobalt cations in spinel oxides to promote the performance of Li–O_2_ battery. Nano Res. (2023). 10.1007/s12274-023-5526-0

[49] Y. Han, J. Dai, R. Xu, W. Ai, L. Zheng et al., Notched-polyoxometalate strategy to fabricate atomically dispersed Ru catalysts for biomass conversion. ACS Catal. **11**, 2669–2675 (2021). 10.1021/acscatal.0c04006

[50] J. Li, H. Zhang, W. Samarakoon, W. Shan, D.A. Cullen et al., Thermally driven structure and performance evolution of atomically dispersed FeN_4_ sites for oxygen reduction. Angew. Chem. Int. Ed. **58**(52), 18971–18980 (2019). 10.1002/anie.201909312 10.1002/anie.201909312 31633848

[51] Y. Zhou, Q. Gu, K. Yin, Y. Li, L. Tao et al., Engineering e_g_ orbital occupancy of Pt with Au alloying enables reversible Li-O_2_ batteries. Angew. Chem. Int. Ed. **61**(26), e202201416 (2022). 10.1002/anie.202201416 10.1002/anie.202201416 35352866

[52] Y. Zhou, Q. Gu, K. Yin, L. Tao, Y. Li et al., Cascaded orbital-oriented hybridization of intermetallic Pd_3_Pb boosts electrocatalysis of Li-O_2_ battery. PNAS **120**(25), e2301439120 (2023). 10.1073/pnas.2301439120 37307482 10.1073/pnas.2301439120 PMC10288638

