# Supplementary material for: Atomically Dispersed Ruthenium Catalysts with Open Hollow Structure for Lithium–Oxygen Batteries
Source: Nanomicro Lett. 2023 Nov 21;16:27. doi: 10.1007/s40820-023-01240-0 (PMC10663429; doi:10.1007/s40820-023-01240-0)
Supplement: Supplementary file 1 — Supplementary file1 (PDF 1847 kb) [file 40820_2023_1240_MOESM1_ESM.pdf]

Supporting Information for

Atomically Dispersed Ruthenium Catalysts with Open Hollow Structure for Lithium–Oxygen Batteries

Xin Chen[1], Yu Zhang[2,]*, Chang Chen[2], Huinan Li[2], Yuran Lin[3], Ke Yu[2], Caiyun Nan[3] and Chen Chen[2,]*

[1] Beijing Advanced Innovation Center for Materials Genome Engineering, Institute of Solid State Chemistry, University of Science and Technology Beijing, Beijing, 100083, P. R. China

[2] Engineering Research Center of Advanced Rare Earth Materials, Department of Chemistry, Tsinghua University, Beijing, 100084, P. R. China

[3] Beijing Key Laboratory of Energy Conversion and Storage Materials Institution, College of Chemistry, Beijing Normal University, Beijing, 100875, P. R. China

*Corresponding authors. E-mail: yuzhangyz@yeah.net (Yu Zhang); cchen@mail.tsinghua.edu.cn (Chen Chen)

Supplementary Figures and Tables

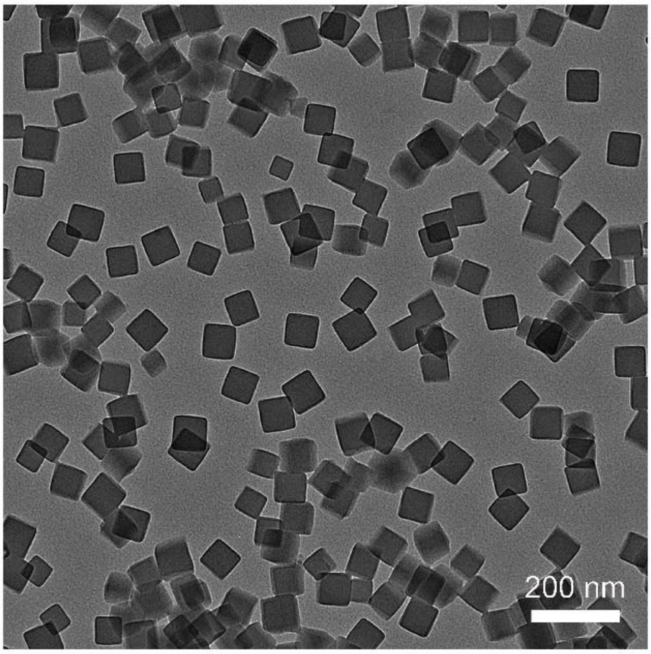

Fig. S1 TEM image of ZIF-8

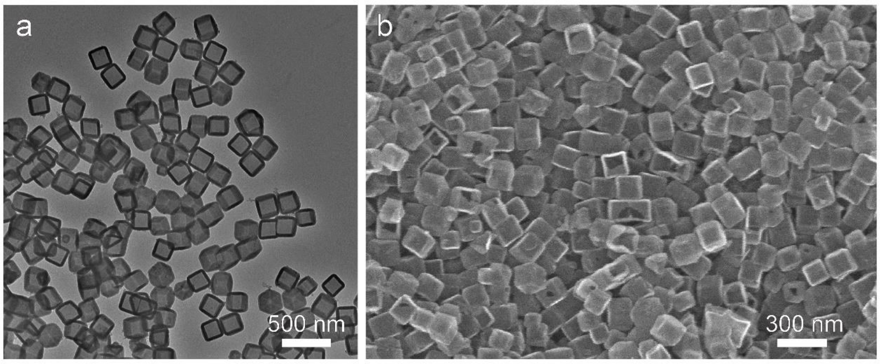

Fig. S2 (**a**) TEM image, (**b**) SEM image of h-ZIF-8

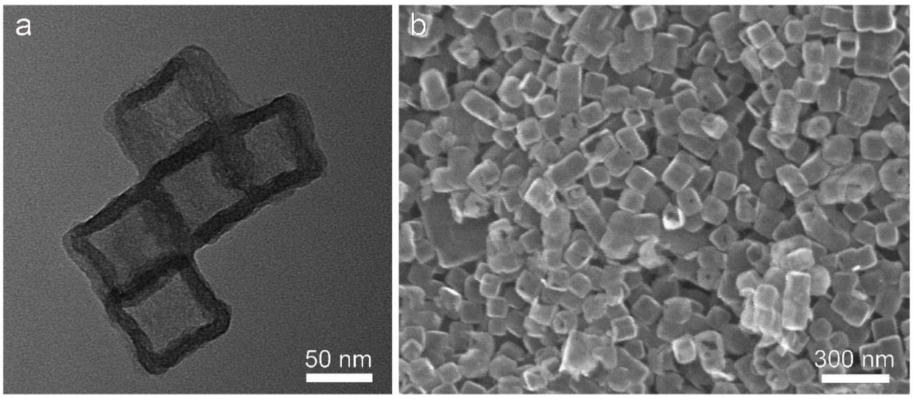

Fig. S3 (**a**) TEM image, (**b**) SEM image of h-NC

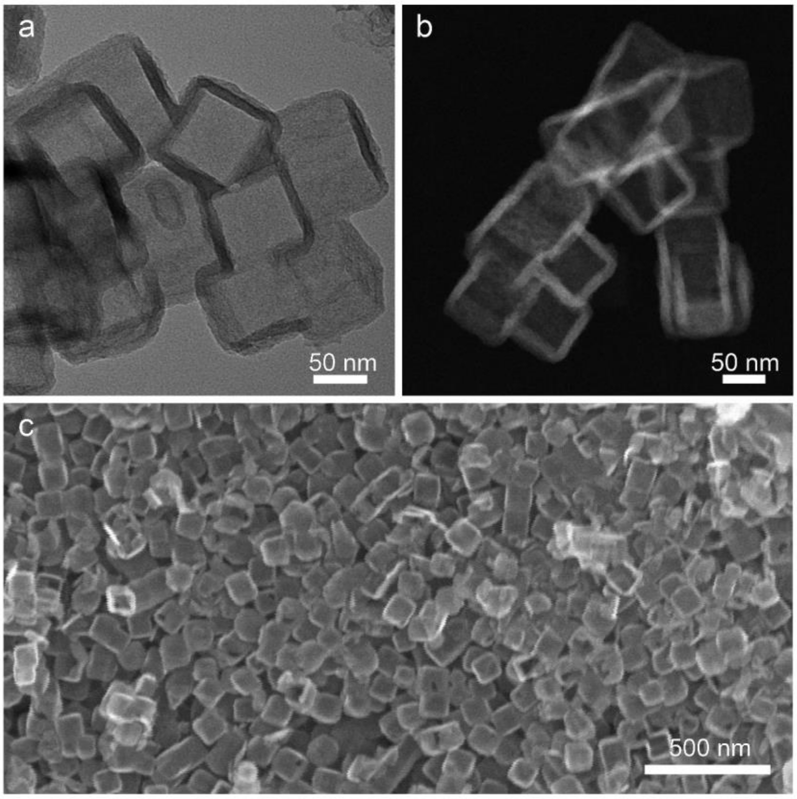

Fig. S4 (**a**) TEM image, (**b**) HAADF-STEM image and (**c**) SEM image of h-RuNC

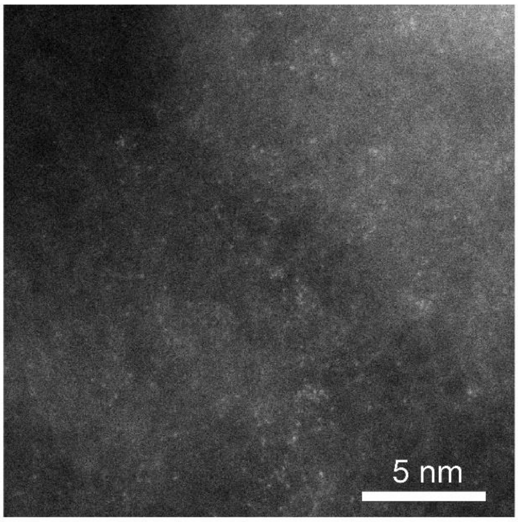

Fig. S5 AC HAADF-STEM image of h-RuNC

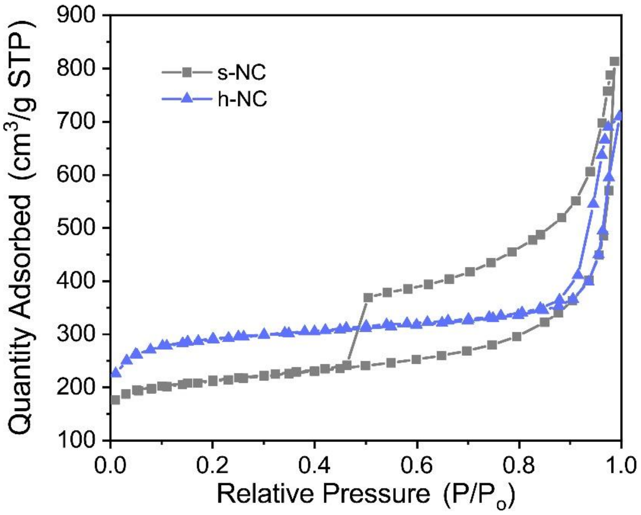

Fig. S6 N_2 adsorption-desorption isotherm of s-NC and h-NC with mesoporous

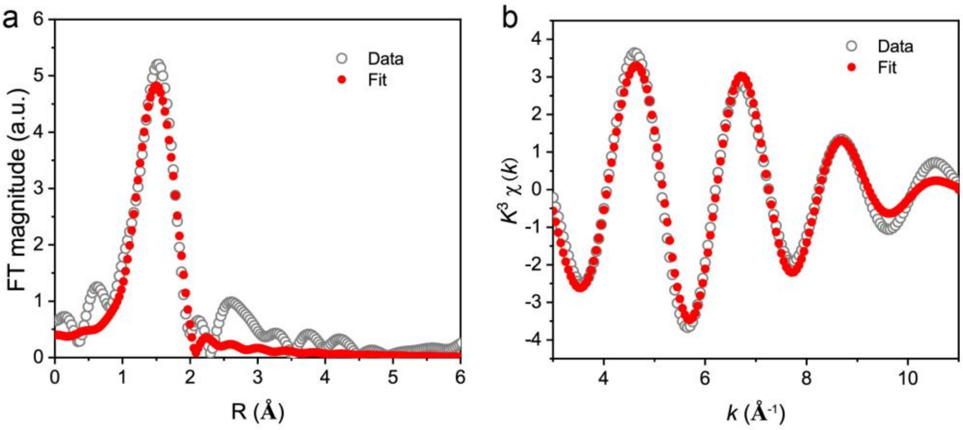

Fig. S7 Ru K-edge EXAFS fitting curves of h-RuNC at R space (**a**) and k space (**b**)

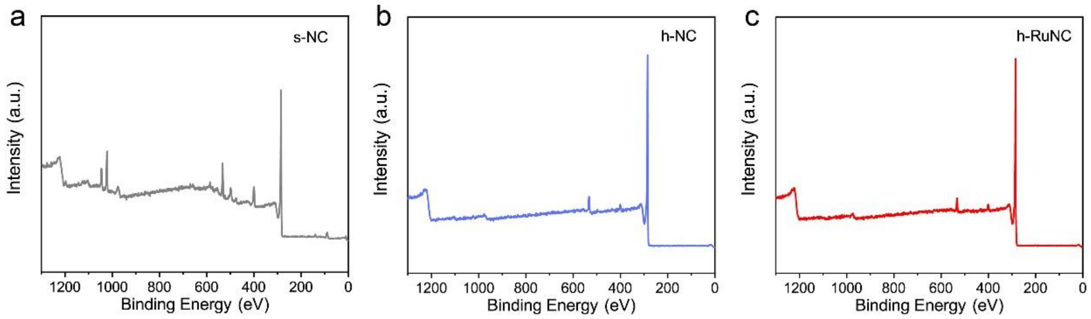

Fig. S8 XPS survey spectra of (**a**) s-NC, (**b**) h-NC and (**c**) h-RuNC

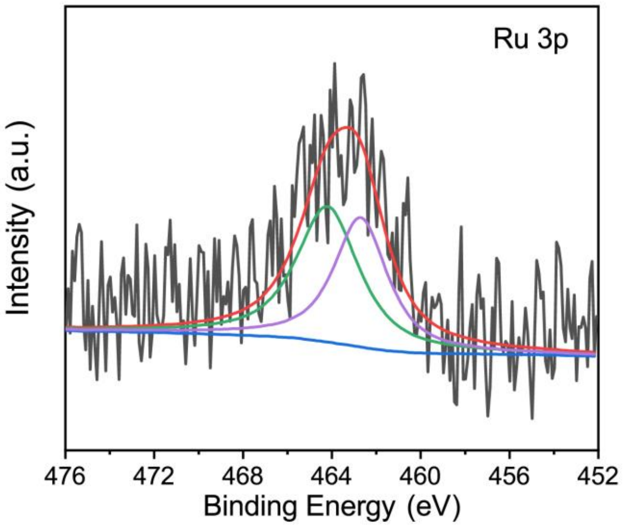

Fig. S9 XPS Ru 3*p* spectrum of h-RuNC

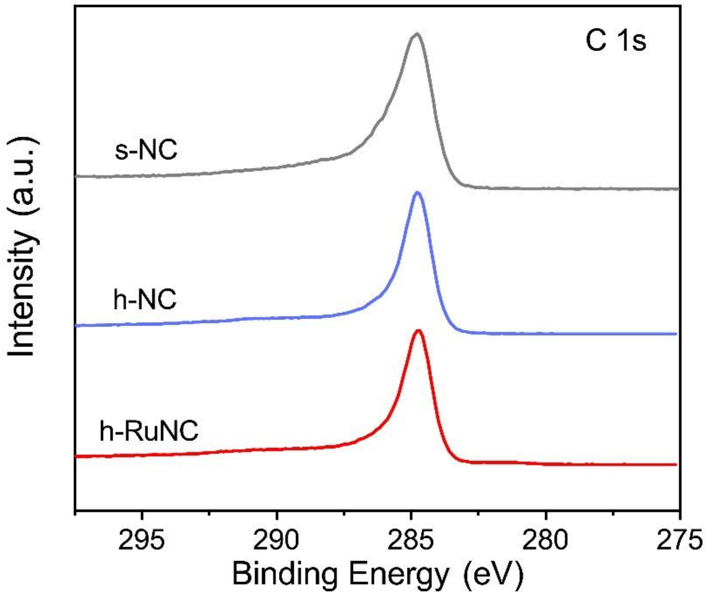

Fig. S10 XPS C 1*s* spectra of s-NC, h-NC and h-RuNC

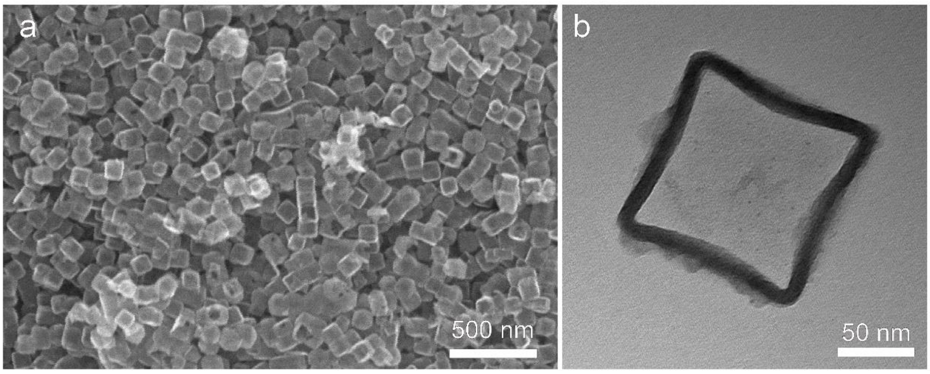

Fig. S11 (**a**) SEM image, (**b**) TEM image of h-$Ru_{NP}NC$

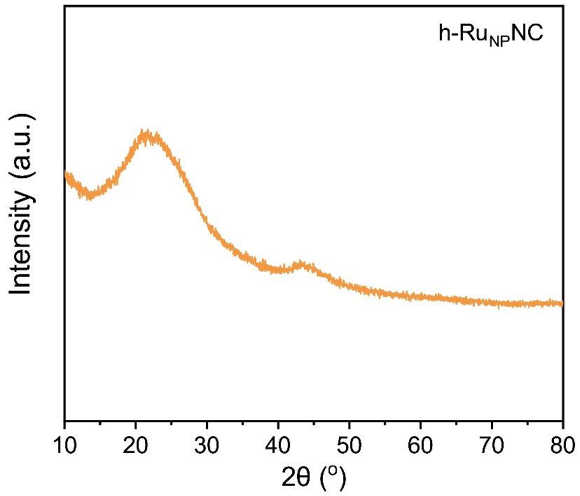

Fig. S12 XRD pattern of h-$Ru_{NP}NC$

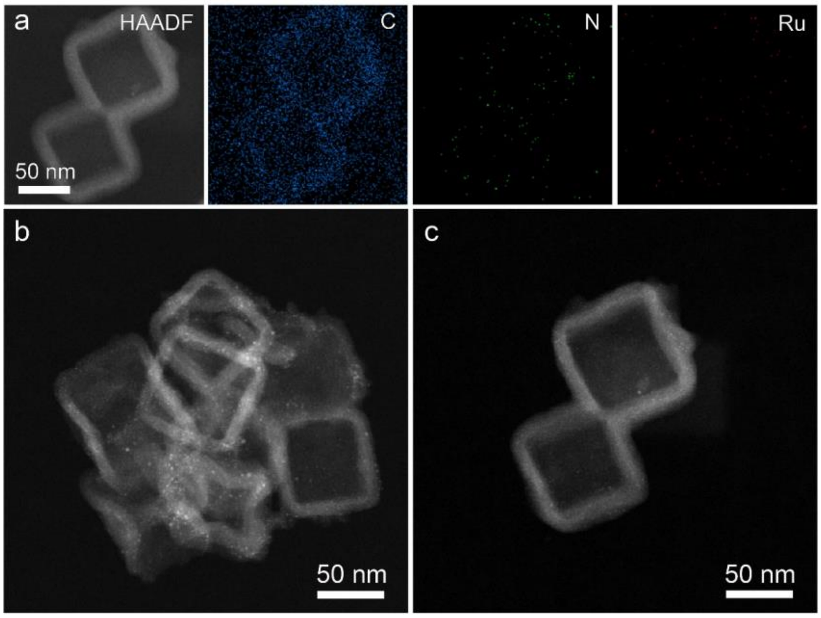

Fig. S13 HAADF-STEM images and corresponding elemental mapping images of h-$Ru_{NP}NC$

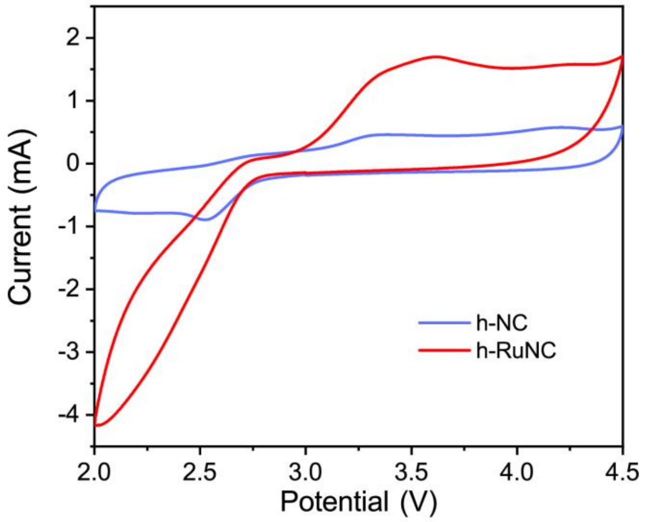

Fig. S14 Cyclic voltammetry curves of h-NC and h-RuNC

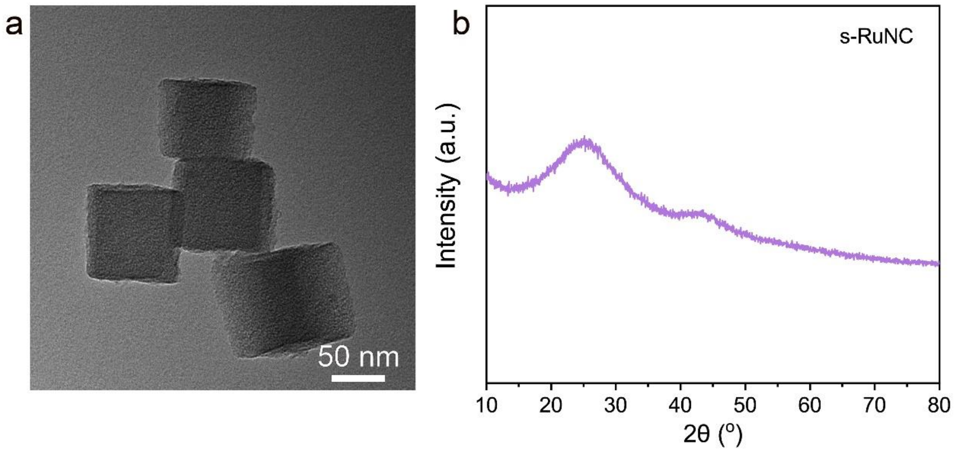

Fig. S15 (**a**) TEM image. (**b**) XRD pattern of s-RuNC

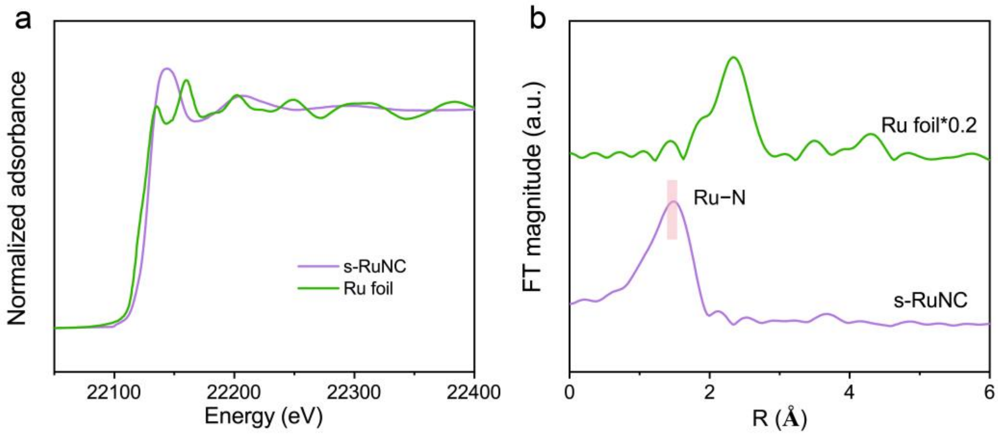

Fig. S16 (**a**) Normalized XANES spectra of Ru K-edge. (**b**) Fourier-transform EXAFS spectra of R space

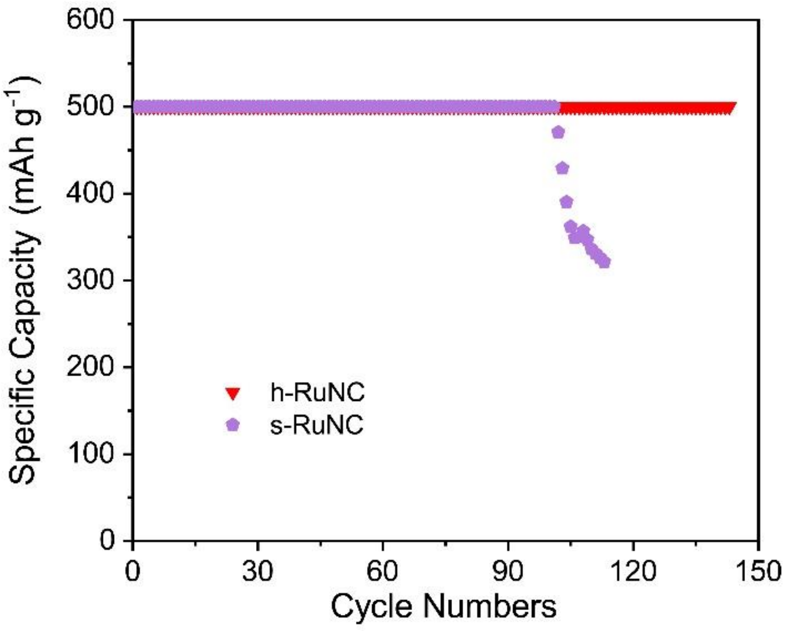

Fig. S17 Cycling performances of h-RuNC and s-RuNC

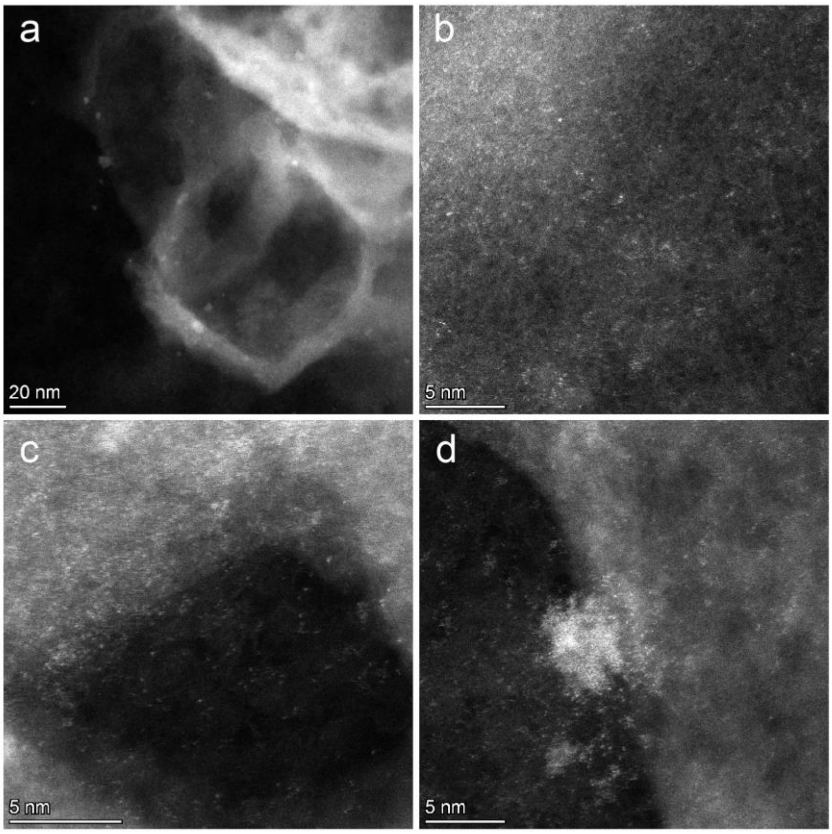

Fig. S18 AC HAADF-STEM image of h-RuNC after 150 cycles

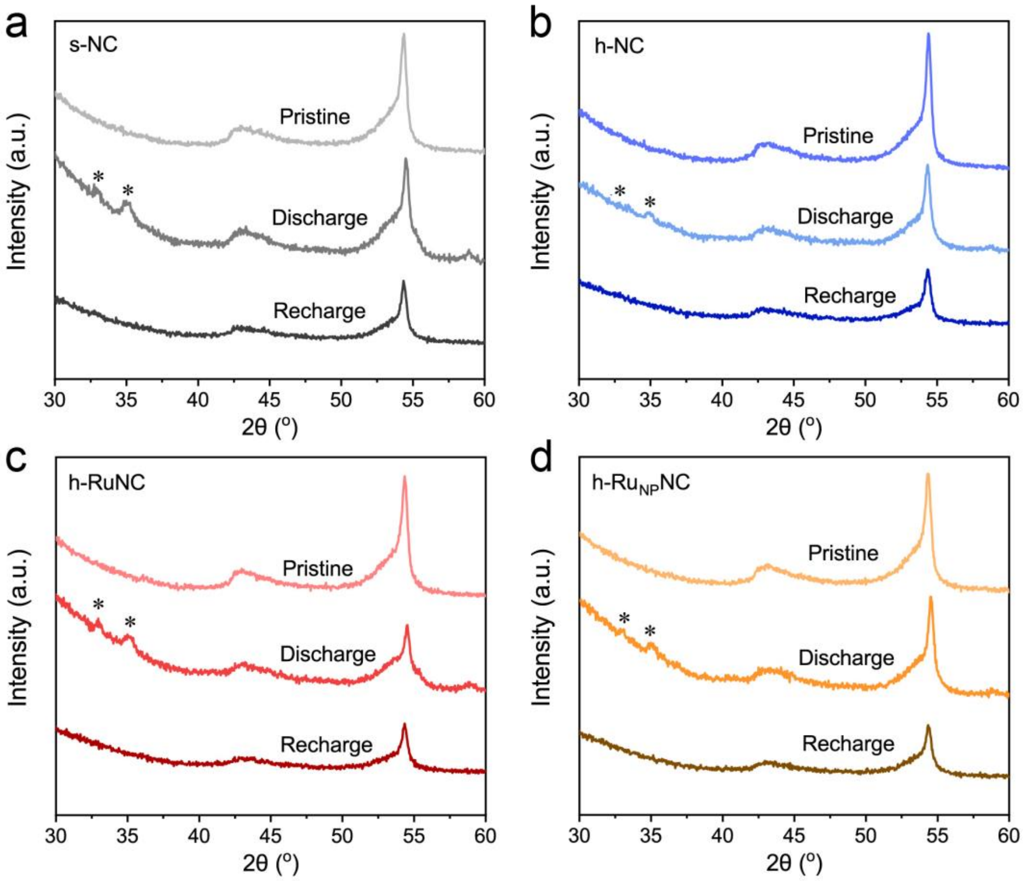

Fig. S19 XRD patterns of s-NC (**a**), h-NC (**b**), h-RuNC (**c**) and h-Ru$_{NP}$NC (**d**) at different state of batteries during discharging and charging processes

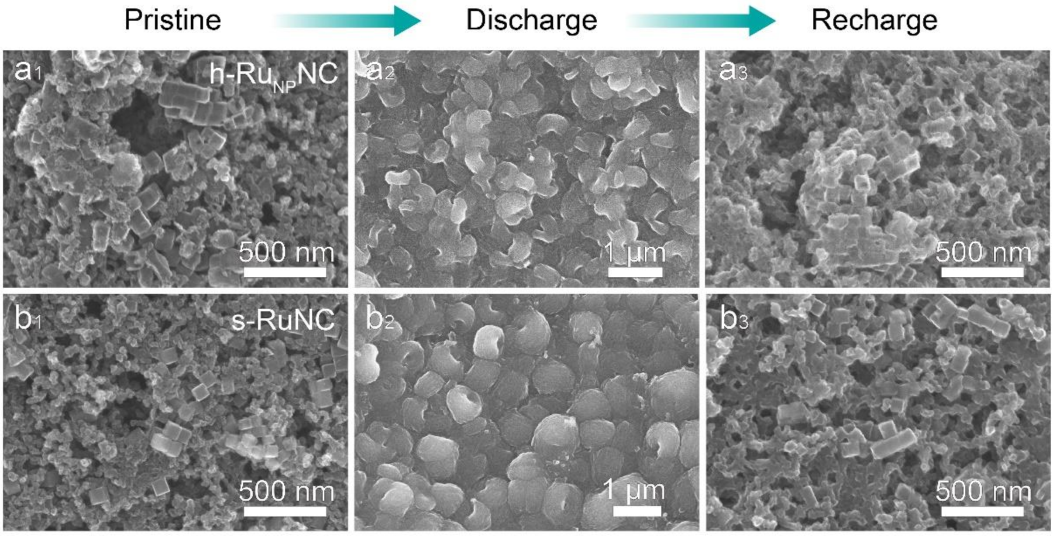

Fig. S20 SEM images of the cathodes at different state based on (**a$_1$-a$_3$**) h-Ru$_{NP}$NC and (**b$_1$-b$_3$**) s-RuNC: (**a$_1$, b$_1$**) pristine, (**a$_2$, b$_2$**) after full discharge, (**a$_3$, b$_3$**) recharge

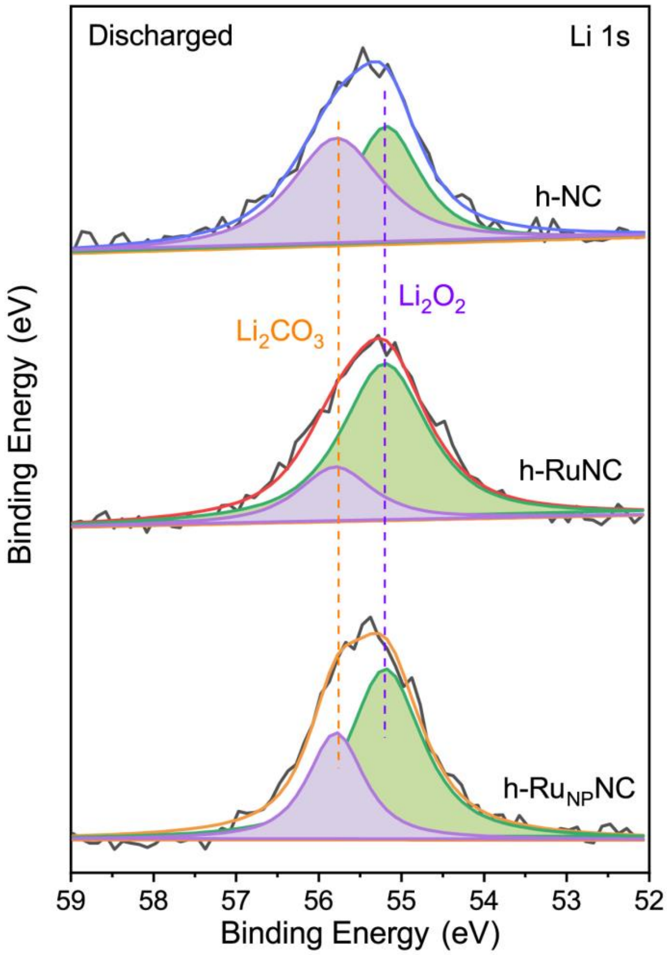

Fig. S21 XPS spectra of h-NC, h-RuNC and h-Ru$_{NP}$NC after discharged

Table S1 Fitting parameters of h-RuNC

Sample	Path	R (Å)	Coordination Number	ΔE_0 (eV)	σ^2 ($Å^2$)	R factor
h-RuNC	Ru-N	1.48(1)	3.9(8)	11.5(4)	0.0093(1)	2.125

Table S2 ICP-OES analysis results of the as-synthesized catalysts

Catalyst	h-RuNC	h-Ru$_{NP}$NC
Ru (%)	1.24	2.70
